# An integrated approach of remote sensing and geospatial analysis for modeling and predicting the impacts of climate change on food security

**DOI:** 10.1038/s41598-023-28244-5

**Published:** 2023-01-19

**Authors:** Mohammad Kazemi Garajeh, Behnam Salmani, Saeid Zare Naghadehi, Hamid Valipoori Goodarzi, Ahmad Khasraei

**Affiliations:** 1 Earth Observation and Satellite Image Applications Laboratory (EOSIAL), School of Aerospace Engineering (SIA), Sapienza University of Rome, Via Salaria 851-881, 00138 Rome, Italy; 2 Department of Remote Sensing and GIS, University of Tabriz, Tabriz, Iran; 3 Department of Civil, Environmental and Geomatics Engineering, College of Engineering and Computer Science, Florida Atlantic University, 777 Glades Road, Boca Raton, FL 33431 USA; 4 Department of Mining Engineering, Isfahan University of Technology, Isfahan, Iran; 5 Department of Irrigation and Drainage, Faculty of Agriculture, Bu-Ali Sina University, Hamedan, Iran

**Keywords:** Climate sciences, Ecology, Environmental sciences

## Abstract

The agriculture sector provides the majority of food supplies, ensures food security, and promotes sustainable development. Due to recent climate changes as well as trends in human population growth and environmental degradation, the need for timely agricultural information continues to rise. This study analyzes and predicts the impacts of climate change on food security (FS). For 2002–2021, Landsat, MODIS satellite images and predisposing variables (land surface temperature (LST), evapotranspiration, precipitation, sunny days, cloud ratio, soil salinity, soil moisture, groundwater quality, soil types, digital elevation model, slope, and aspect) were used. First, we used a deep learning convolutional neural network (DL-CNN) based on the Google Earth Engine (GEE) to detect agricultural land (AL). A remote sensing-based approach combined with the analytical network process (ANP) model was used to identify frost-affected areas. We then analyzed the relationship between climatic, geospatial, and topographical variables and AL and frost-affected areas. We found negative correlations of − 0.80, − 0.58, − 0.43, and − 0.45 between AL and LST, evapotranspiration, cloud ratio, and soil salinity, respectively. There is a positive correlation between AL and precipitation, sunny days, soil moisture, and groundwater quality of 0.39, 0.25, 0.21, and 0.77, respectively. The correlation between frost-affected areas and LST, evapotranspiration, cloud ratio, elevation, slope, and aspect are 0.55, 0.40, 0.52, 0.35, 0.45, and 0.39. Frost-affected areas have negative correlations with precipitation, sunny day, and soil moisture of − 0.68, − 0.23, and − 0.38, respectively. Our findings show that the increase in LST, evapotranspiration, cloud ratio, and soil salinity is associated with the decrease in AL. Additionally, AL decreases with a decreasing in precipitation, sunny days, soil moisture, and groundwater quality. It was also found that as LST, evapotranspiration, cloud ratio, elevation, slope, and aspect increase, frost-affected areas increase as well. Furthermore, frost-affected areas increase when precipitation, sunny days, and soil moisture decrease. Finally, we predicted the FS threat for 2030, 2040, 2050, and 2060 using the CA–Markov method. According to the results, the AL will decrease by 0.36% from 2030 to 2060. Between 2030 and 2060, however, the area with very high frost-affected will increase by about 10.64%. In sum, this study accentuates the critical impacts of climate change on the FS in the region. Our findings and proposed methods could be helpful for researchers to model and quantify the climate change impacts on the FS in different regions and periods.

## Introduction

The impacts of climate change can be seen globally, regionally, and nationally in all sectors of the economy such as agriculture, construction, tourism, energy, health and productivity^1^. They threaten traditional agriculture, livestock, forestry, and the infrastructure of local communities^2,3^. Agriculture is a key responsibility of climate change, which follows strong seasonal patterns linked to the biological life cycle of crops and rangelands, and is also affected by climatic factors and physical characteristics of the landscape^4,5^. Across the globe, climate change impacts agriculture through limited rainfall, high temperatures, and infestations of noxious pests and diseases^6^. Temperature and precipitation are monitored to determine how climate change affects agricultural production^7,8^. As noted in the Intergovernmental Panel on Climate Change (IPCC) Climate Change and Land report, climate changes such as increased rainfall, temperature changes, and water scarcity adversely affect agricultural productivity^9^.

Agricultural production accounts for the largest share of food supplies and provides several ecosystem services (e.g., food provisioning)^10^. Agriculture is responsible (directly or indirectly) for about 90% of calories in food and 80% of proteins and fats (livestock production)^11^. Hence, agriculture is vital to food security and sustainable development goals^12,13^. Massive efforts have been made to increase agriculture food production and security since the 1996 World Food Summit (WFS)^14,15^. Despite the great efforts made over the last decades toward attaining global food security, one in ten people worldwide suffers from severe food insecurity^16,17^. Growing population, accelerated urbanization, non-sustainable consumption of non-renewable resources, climate change, and changing food consumption patterns (e.g., more calories consumed; changes in diet structure toward meat and eggs, among other things), will pose serious challenges to food security^18,19^.

In the most vulnerable regions with weakened agricultural systems, climate change will negatively impact crop yields and, eventually, food supply and accessibility^20^. Considering these changing situations and the negative outlook, it is of utmost importance to monitor and provide reliable predictions of climate change impacts to better allocate scarce resources, design early warning systems, and maintain food security. In order to minimize the trade-offs associated with climate change impacts on food supply, it is crucial to invest and develop new technologies for data acquisition (e.g., remote sensing and proximal sensing) as well as create robust and validated models, which are derived data from multiple sources^21,22^. By doing this, we will be able to identify more accurately which climate change factors are more severe and where can have more adverse effects on ecosystems^23^. The management of agricultural land will be improved as a result of this. In order for this to be fully operational, interdisciplinary research is required^24,25^. Agriculture can greatly benefit from remote sensing since it allows gathering information about the biophysical state of vegetation over large areas with frequent revisits^26^. Using remote sensing techniques, large amounts of granular information can be collected at different spatio-temporal resolution scales over large areas^27^. Through analyzing natural features on the ground and monitoring their changes over time, remote sensing applications will ultimately aid policy-making in the food sector at different levels^28,29^. Traditionally, crop yield estimates were based on farmers' field survey data collected during the growing season. In addition to being time-consuming, they are hard to scale up to larger areas^30,31^. Several studies have used remote sensing-based indexes such as the normalized difference vegetation index (NDVI) and enhanced vegetation index (EVI) for agricultural production assessment^32–34^. These indicators, however, are affected by atmospheric and earth factors since they rely on spectral reflections of the earth's features^35^. In 2001, the Monitoring Agricultural Resources unit (MARS) began developing techniques for agricultural production in areas with high food insecurity risks in Europe (to provide early warning information about the FS)^36^. FS-based assessment projects have also been conducted since 2006 for Ethiopia, East and West Africa, Niger, North Korea, Angola, and others^37^. A new early warning system called ASAP (Anomaly Hot Spots of Agricultural Production) was recently developed and launched by MARS. Time series analysis based on models, remote sensing observations, and well-established time series datasets is used in ASAP to analyze global climate and vegetation data. As input to more detailed agricultural monitoring or food security assessments, the system provides timely overviews of production anomalies at the global level^38,39^. However, the proposed system is not available for all regions around the world. Additionally, it considers drought-related production deficits using both indicators (Rainfall rate and NDVI) in countries with negative annual climatic water balances (for example, precipitation—potential evapotranspiration). As mentioned before, using remote sensing-based indices would adversely affect the accuracy of obtained datasets due to atmospheric and earth’s features. This system also fails to provide a practical framework for assessing the impacts of frost on agricultural production, which has been increasing during the last decades following the impacts of climate change^40–43^. Land use/cover (LULC) change is one of the most important factors to evaluate the effects of climate change on food security^44,45^. Satellite imagery has been extensively used by scientists to map and monitor the impacts of climate change on food security based on LULC changes^35,38–43,46,47^.

Through the application of learning-based statistical algorithms, new frontiers have been opened for the analysis of satellite imagery, which provides better and more nuanced insights due to their ability to identify patterns behind the complex nonlinear relationships that characterize environmental variables^48^. Probabilistic machine learning has demonstrated the importance of quantifying uncertainty with any statement about the future to provide a full picture of the possible scenarios and to inform decision-making at all levels^49–53^. Furthermore, the availability of cloud-free, high-quality images and the utility of multi-temporal, high-resolution datasets over large areas have been overcome through a paradigm shift in remote sensing data collection, processing and management^54^. Utilizing powerful machine learning algorithms in cloud computing environments such as Google Earth Engine (GEE), a multi-petabyte archive of georeferenced datasets can be combined in the GEE catalog, which includes images from earth-observing satellites and airborne sensors, weather and climate datasets, as well as digital elevation models^55^. GEE provides computing and storage resources, and major machine learning algorithms useful for image enhancement and classification, with batch processing available through JavaScript and Python on Application Program Interfaces (APIs)^56,57^. As a result, most of the preprocessing steps that are required in traditional remote sensing approaches are reduced^55^.

Literature review shows that relatively few studies explore the impacts of climate change on the FS using an integrated approach of remote sensing, deep learning convolutional neural network (DL-CNN) and GEE. Furthermore, previous studies have mostly focused on the effects of climate change on LULC. However, this research also has the advantage of modeling the effects of frost on garden products, one of the main sources of food supplies in Lake Urmia Basin (LUB). They also used remote sensing-based indexes (e.g., NDVI) for crop yield estimates to assess the FS, which are based on the reflectance of the earth’s features. Finally, while previous studies had only considered a few predisposing variables (e.g., temperature and precipitation) for modelling the effects of climate change on the FS, this research aims to include more climatic (e.g., evapotranspiration), geospatial (e.g., groundwater quality), and topographical (e.g., slope) variables. Therefore, this study aims to (1) examine the effects of climate change on the FS in the LUB during the period 2002–2021, (2) evaluate the effectiveness of different climatic, geospatial, and topographical variables on the FS, and (3) predict the impacts of climate change on the FS over the study area for 2030, 2040, 2050, and 2060 using CA–Markov model.

## Materials and methodology

### Materials

Landsat series images with a spatial resolution of 30 m as well as climatic, geospatial, and topographical variables were used to model the impacts of climate change on the FS. The cloud-free images were obtained from www.glovis.com for 6th and 13th September 2002, 11th and 18th August 2010, 24th August and 2nd September 2015, and 24th August and 9th September 2021. Various predisposing variables, including climatic, geospatial as well as topographical were acquired from different sources (Table 1) for dried months that the impact of climate change are more sever, which will be explored in the following sections:

**Table 1:** Characteristics of predisposing variables used to assess the impact of climate change on the FS.

Category	Variable	AL	Frost	Spatial resolution	Coordinate system	Data source
Satellite image	Landsat 5, 7 and 8	*	–	30 m	WGS^a^ 1984-UTM^b^-Zone 35	www.glovis.com
Climatic	LST	–	*	30 m	WGS 1984-UTM-Zone 35	Landsat images
	ET	–	–	1 km	WGS 1984-UTM-Zone 35	MOD16 ET datasets
	Precipitation	–	*	1 km	WGS 1984-UTM-Zone 35	Monthly Global Precipitation Measurement (GPM)
	Sunny days	–	*	1 km	WGS 1984-UTM-Zone 35	Meteorological station
	Cloud ratio	–	*	1 km	WGS 1984-UTM-Zone 35	Meteorological station
Geospatial	Soil salinity	–	–	30 m	WGS 1984-UTM-Zone 35	Landsat images
	Soil moisture	–	*	1 km	WGS 1984-UTM-Zone 35	Monthly Climate and Climatic Water Balance for Global Terrestrial Surfaces datasets
	Groundwater quality	–	–	1 km	WGS 1984-UTM-Zone 35	https://eaj.ir
	Soil types	–	*	1000 km	WGS 1984-UTM-Zone 35	Iran National Soil Texture Map
Topographical	DEM	–	*	12.5 m	WGS 1984-UTM-Zone 35	www.vertex.alaska.edu
	Slope	–	*	12.5 m	WGS 1984-UTM-Zone 35	DEM
	Aspect	–	*	12.5 m	WGS 1984-UTM-Zone 35	DEM

^a^World Geodetic System.

^b^Universal Transverse Mercator.

## Climatic variables

### Land surface temperature (LST)

**Figure 1:**
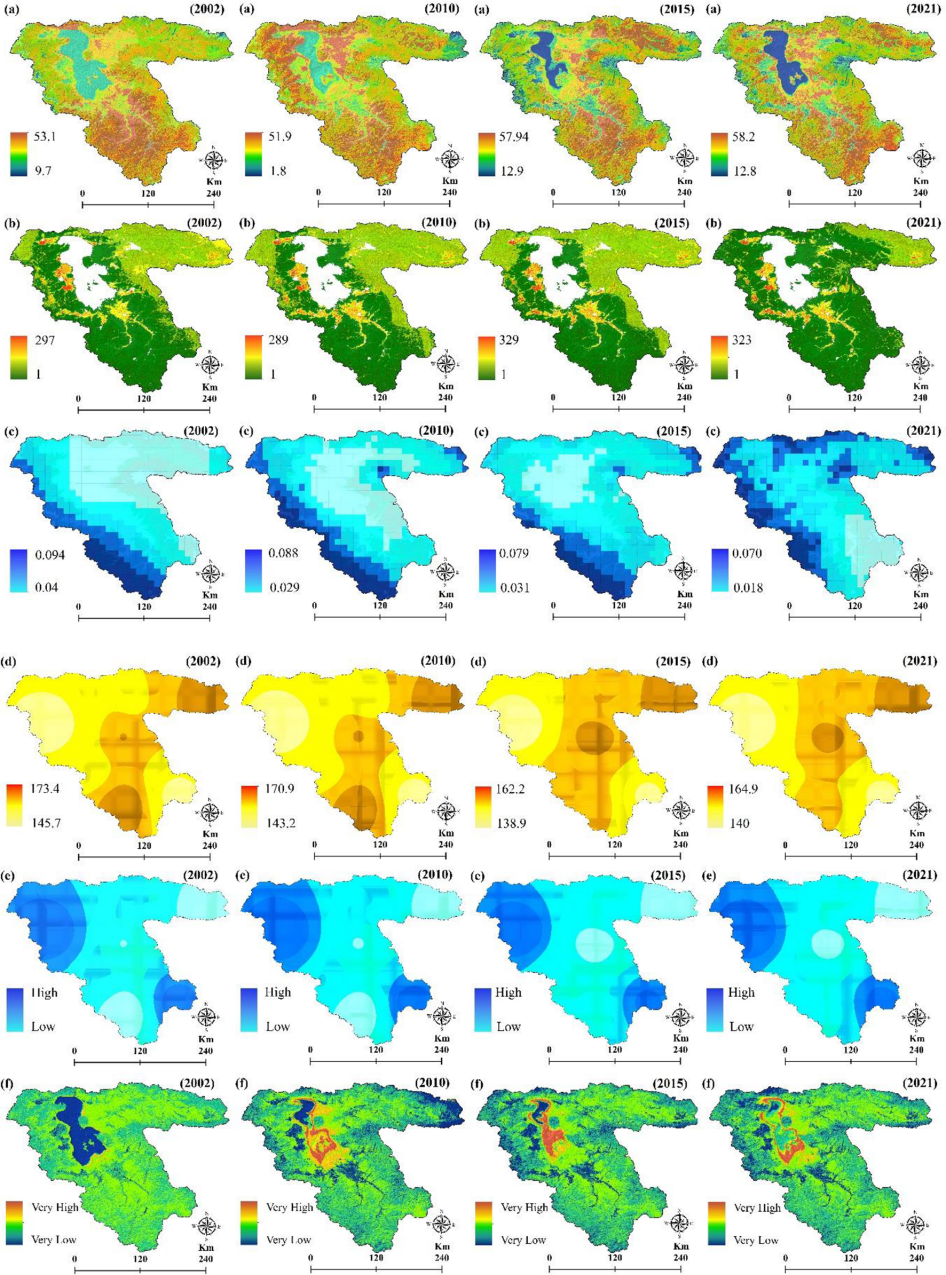

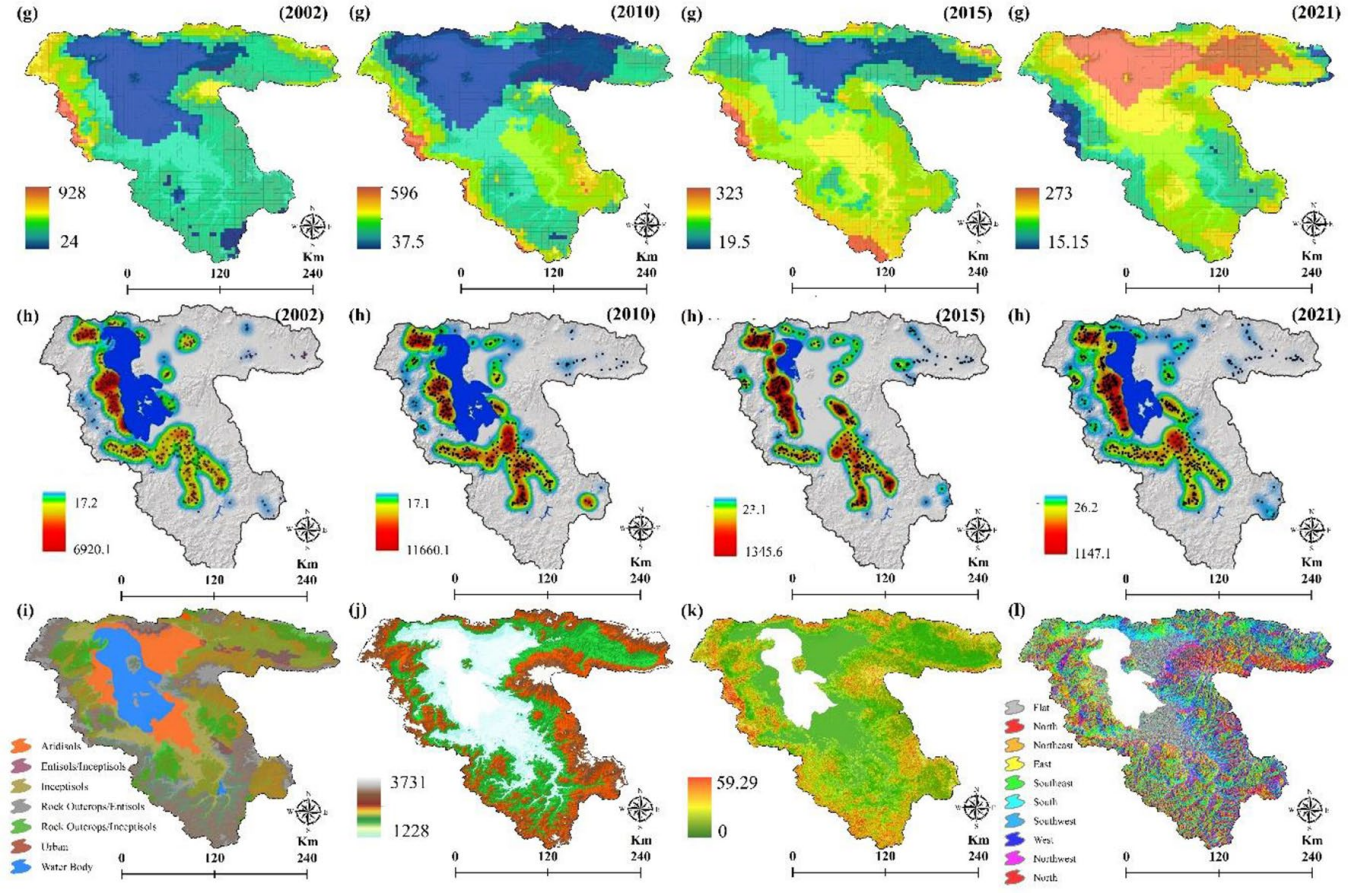
Predisposing variables for assessing the impacts of climate change on the FS, generated in the ArcGIS 10.6 software (www.esri.com); (**a**) LST (°C) for years 2002, 2010, 2015, and 2021, (**b**) ET in milliliter for years 2002, 2010, 2015, and 2021, (**c**) precipitation for years 2002, 2010, 2015, and 2021, (**d**) sunny days for years 2002, 2010, 2015, and 2021, (**e**) cloud ratio in percentage for years 2002, 2010, 2015, and 2021, (**f**) soil salinity distribution for years 2002, 2010, 2015, and 2021, (**g**) soil moisture for years 2002, 2010, 2015, and 2021, (**h**) groundwater quality for years 2002, 2010, 2015, and 2021, (**i**) soil types, (**j**) DEM, (**k**) slope in degree, and (**l**) aspect.

The effects of climate change on the environment include an increase in rainfall intensity, a rise in the LST, and long-term droughts. The LST is a crucial parameter in land surface physics, which can significantly affect the FS^2^. For the study area, LST is estimated from Landsat Thematic Mapper (TM), Enhanced Thematic Mapper (ETM +) (Band 6) and Thermal Infrared Sensor (TIRS) (Band 10) images in the GEE platform for the years 2002, 2010, 2015, and 2021 with Root Mean Square Error (RMSE) of 2.01, 2.18, 1.55, and 1.91, respectively (Fig. 1a). Equation (1) is used to estimate the spectral radiance and Eq. (2) is accordingly applied to retrieve the LST^58^:

1 $${L}_{\lambda }={G}_{rescale}\times {Q}_{cal}+{B}_{rescale}$$

where ${L}_{\lambda }$ is the spectral radiance at the sensor aperture $W/({m}^{2}sr \mu m)$, ${Q}_{cal}$ is the quantized calibrated pixel value in digital number, ${G}_{rescale}$ is the band-certain rescaling gain factor $W/({m}^{2}sr \mu m)/DN)$, and ${B}_{rescale}$ is the band-specific rescaling bias factor $W/({m}^{2}sr \mu m))$.

2 $$T={K}_{2}/In(({K}_{1}/{L}_{\lambda })+1)$$

where $T$ is LST, ${K}_{1}$ is constant values for B6 of ETM + (666.09) and B10 of TIR (774.89), ${K}_{2}$ is constant values for B6 of ETM + (1282.71) and B10 of TIR (1321.08), which represent calibration coefficients, and ${L}_{\lambda }$ is radiated images.

### Evapotranspiration (ET)

On average, evaporation contributes 60–65% of the average precipitation over the land surface of the Earth, making it the second most significant component of the hydrological cycle after precipitation. The increasing human population and recent climatic variations have increased the importance of quantifying evaporation^59^. This study used the MOD16 (MODIS) global evapotranspiration (ET) products to analyze the impact of climate change on the FS (Fig. 1b). The MOD16 ET datasets are estimated using^60^. The ET algorithm is described as Eq. (3)^61^.

3 $$\lambda E=\frac{S\times A+\rho \times {C}_{p}\times ({e}_{sat}-e)/{r}_{a}}{s+\gamma \times (1+{r}_{s}/{r}_{a})}$$

where $\lambda E$ denotes the latent heat flux and $\lambda$ represents the latent heat of evaporation; $s=d({e}_{sat})/dT$, the slope of the curve associated with saturated water vapor pressure $({e}_{sat})$ to temperature. $A$ represents available energy divided between sensible heat, latent heat as well as soil heat fluxes on the ground. $\rho$ represents air density, ${C}_{p}$ denotes the certain heat capacity of air, and ${r}_{a}$ represents the aerodynamic resistance. The psychometric constant $\gamma$ is assigning through $\gamma ={C}_{p}\times {P}_{a}\times {M}_{a}/(\lambda \times {M}_{w})$, where ${M}_{a}$ and ${M}_{w}$ represent the molecular masses of arid air and wet air, respectively. ${P}_{a}$ denotes atmospheric pressure. ${r}_{s}$ is an efficient resistance to evaporation from land surface and transpiration from the plant canopy.

### Precipitation

Trees become more resistant to frost when rainfall increases. Frost resistance will increase with higher rainfall during the flowering and fruit production season^62^. We used monthly and daily precipitation datasets collected from Monthly Global Precipitation Measurement (GPM) v6^63^ and East Azerbaijan Meteorological Organization for the years 2002, 2010, 2015, and 2021(Fig. 1c).

### Sunny days

Trees require sunlight to increase their productivity. As a result, frost risk decreases as it increases^64^. This study used climatic datasets to generate sunny days maps collected from the East Azerbaijan Meteorological Organization from 2002 to 2021 (http://eamo.ir) (Fig. 1d).

### Cloud ratio

In cases where there is a thick cloud layer, the radiation contrast between night and day is balanced, thereby increasing the minimum and decreasing the maximum temperature. Therefore, temperature fluctuations or the difference between the maximum and minimum temperatures are much smaller on cloudy days than on clear ones^65^. In this study, we employed climatic datasets collected from the East Azerbaijan Meteorological Organization to generate cloud ratio maps for the years 2002, 2010, 2015, and 2021 (Fig. 1e).

## Geospatial variables

### Soil salinity

In semi-arid and arid regions, soil salinity is a major and widespread threat to the FS and the environment. This study used the SI3 index (Eq. 4) based on Landsat series images to estimate soil salinity throughout the study area from 2002 to 2021, which its efficiency for soil salinity estimation was emphasized by^24^ (Fig. 1f).

4 $$SI3=[{(R)}^{2}+{(G)}^{2}{]}^{0.5}$$

where $R$ is band red, and $G$ is band green of Landsat images.

### Soil moisture

Plants are directly and indirectly affected by soil moisture since it increases ET and water stress^66^. To produce soil moisture maps for the years 2002, 2010, 2015, and 2021, we used TerraClimate: Monthly Climate and Climatic Water Balance for Global Terrestrial Surfaces datasets received from the University of Idaho (https://data.nkn.uidaho.edu) (Fig. 1g).

### Groundwater quality

Lake Urmia Basin’s (LUB) agricultural sector relies heavily on groundwater. We assessed more than 856 wells in the LUB from a chemical perspective (Fig. 1h). According to the groundwater level simulation, agriculture and industry have withdrawn at least 5–15 m of groundwater, which represents a decline of about 50–60% over the past 3 decades.

### Soil types

There is a temperature difference of 1.7 degrees Celsius depending on the type of soil surface. The amount of energy transferred from the soil to plants depends on the amount of heat stored in the soil. Various soil characteristics, such as soil moisture, soil type, soil color, and the type of ground cover, affect how much heat is stored in the soil during frost. Darker soil absorbs more heat during the day, while lighter soils have a higher albedo and therefore absorb less. The temperature difference between soils in different regions can play an important role in intensifying or weakening production^65^. The soil type map of the study area was obtained from the Iran National Soil Texture Map at a scale of 1:1,000,000 (http://www.swri.ir) (Fig. 1i).

## Topographical variables

### Digital Elevation Model (DEM)

Altitude classes are among the general characteristics of the region, where the lowest annual temperature coincides with the peaks of the mountains. The hottest part is at the bottom. Frostbite is influenced by height, and floors with a high height have a greater value compared to floors with a low height^67^. The DEM was derived for free from www.vertex.alaska.edu with a spatial resolution of 12.5 m (Fig. 1j).

### Slope

Wind volume and velocity are affected by the slope at any given time. A steep slope can lead to higher velocity and, accordingly increase the volume of cold air. In this study, the slope map was acquired from the DEM (Fig. 1k).

### Aspect

The aspect plays a major role in the horizontal distribution of temperature, for example, in the northern hemisphere, the southern slopes of hills and mountains receive more direct radiation from the sun. If the northern slopes are less exposed to the sun. The southwest slopes are warmer than the southeast slopes. Because in the early morning in the southeast slopes, the air is cold and most of the radiant energy is used to heat the air. The biggest temperature difference between the northern and southern slopes is in the spring and summer months. In spring, the southern slopes heat up quickly, while the northern slopes remain cold and humid. We used DEM to derive an aspect map of the study area (Fig. 1l).

## Methodology

**Figure 2:**
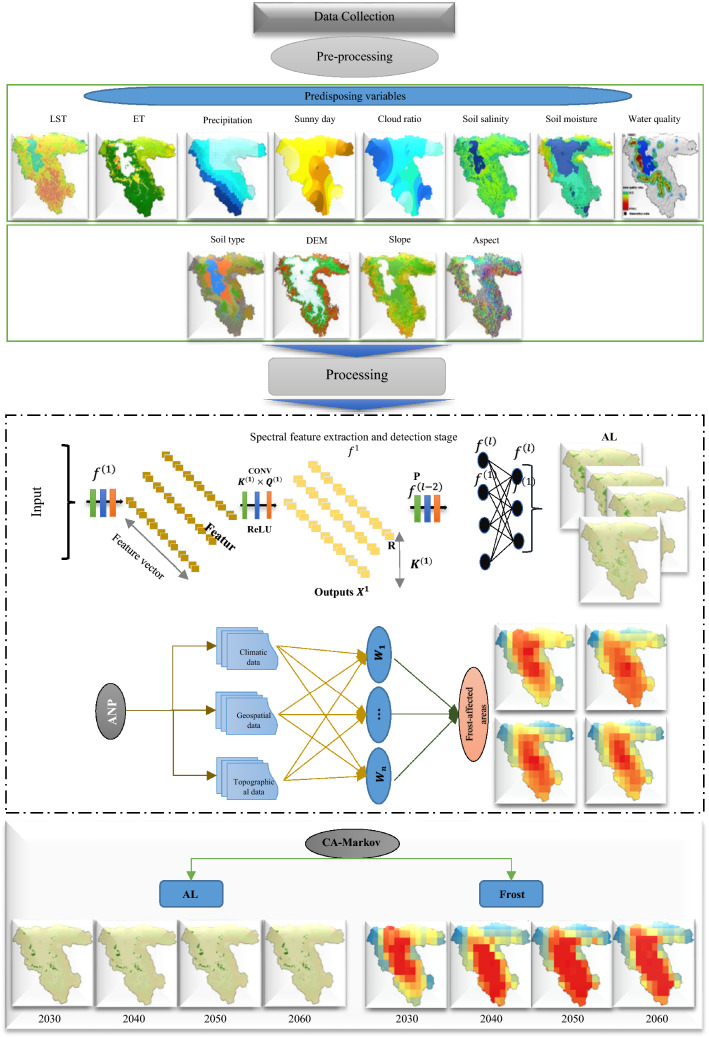
An overview of the presents study methodology.

For FS assessment, mapping AL and frost-affected areas’ patterns are critical. There are four steps in our methodology. The first step is data preparation, which consists of pre-processing climatic (e.g., LST), geospatial (e.g., soil salinity), and topographic (e.g., slope) datasets. This study was evaluated frequent values of LST, ET, precipitation, sunny days, cloud ratio, soil salinity, soil moisture, and groundwater quality from 2002 to 2021. Increase or decrease in these variables would be a sign of climate change impact. The second step is to use DL-CNN and ANP methods to detect and map AL and frost-affected areas using various predisposing variables (e.g., climatic, geospatial and topographic), respectively. In this regard, increase or decrease in the areas of AL and frost can emphasize the effects of climate change. Third, we predicted the impacts of climate change on the FS using the CA–Markov model once our targeted maps were prepared. As a final step, we analyzed the relationships between the predisposing variables and the FS. The FS modeling and mapping methodology is summarized in Fig. 2.

### Deep learning convolutional neural network for AL mapping

AL patterns and changes are closely connected to human activities and profoundly impact the FS^68^. As mentioned in the research literature, DL-CNN has performed well for LULC detection and mapping. The DL-CNN algorithm has been emphasized for its ability to address the difficulties associated with traditional artificial neural networks (ANNs), such as high redundancy generated by the existence of a large number of hidden layers and incapacity in processing large datasets^69^. This is due to the structure of its input and hidden layers, which consist of neuron layers that contain width, height, and depth dimensions for better handling large numbers of multi-channel and large image sets^47^. This study employed an automated DL-CNN with 978 ground control points (GCPs) based on GEE for agricultural land mapping. By analyzing the relationship between climatical and geospatial variables and AL changes, the impacts of climate change can be monitored. Identifying the impact of climate change on the FS using remote sensing data is possible if long enough time series of images are available. The medium spatial–temporal resolution of Landsat images with a revisit frequency of maximum 16 days, is especially well suited for identifying LULC and for studying LULC dynamics.

### Hardware and software

Keara, a Python package based on TensorFlow in the GEE platform was used to build and train the DL-CNN model to detect and map AL. The computer system specifications employed were Intel Core i7-6700 K, VGA (GTX 1080), HDD (256 GB SSD + 1 TB SATA) and 32 GB memory. All the prediction models were implemented under the Python programing language. An open-source high-level Keras library was employed to construct the DL-based AL models.

### Training phase

To map the AL patterns from 2002 to 2021, a set of convolutional layers was used to train our DL-CNN models for the AL classification. Each convolutional layer involves several important factors, including a pooling operation, multiple weights, and an activation function. As part of the encoder, max-pooling was used with $2\times 2$ filters and a two-pixel stride to down-sample the feature maps using a maximum operator by taking the maximum of each $4\times 4$ matrix. This study used a $256\times 256$ pixel input window to detect and map AL.

An activation function is a function that is added to an artificial neural network in order to help the network to learn complex patterns^70^. Different activation functions exist (e.g., sigmoid, softmax, tanh, hyperbolic tangent, ReLU and Leaky ReLU), which are required for forward propagation and its derivative for back propagation. Sigmoid, tanh, hyperbolic tangent, and Softmax are typically used in normal neural networks. On the other hand, Rectified Linear Units (ReLu) are commonly used in CNN algorithms since they perform better^71^. Equation (5) defines the ReLU parameters as follows:

5 $$f(x) = \left\{ {\begin{array}{*{20}c} {xifx > 0} \\ {0ifx \le 0} \\ \end{array} = {\text{max}}\;{\text{(x,0)}}} \right.$$

where 0 is negative input and $x$ is positive output of variables ($x$).

The loss function is a fancy mathematical term for an object that measures how often a model makes an incorrect prediction. In the context of classification, they measure how often a model misclassifies members of different groups^72^. There are several loss/cost functions for problem-solving in classification, including Mean Squared Error (MSE), Cross-Entropy, and Mean Absolute Error (MAE). This study used cross-entropy to measure the performance of a classification model, which is a popular function for problems requiring binary classification^68^ (Eq. 6).

6 $$L\left(y, \widehat{y}\right)=-\frac{1}{N}\sum_{i=1}^{N}\left({y}_{i}\mathrm{log}\left({\widehat{y}}_{i}\right)\right)+\left(1-{y}_{i}\right)\mathrm{log}(1-{\widehat{y}}_{i}))$$

where $N$ is the number of sample datasets, ${y}_{i}$ iss the actual output of ample $i$, which is equals to 0 or 1, ${\widehat{y}}_{i}$ is the forecasted possibility sample $i$ having output 1, and ${y}_{i},{\widehat{y}}_{i}$ are the vectors of actual outputs and forecasted possibilities.

A final objective of forwarding propagation and back-propagation is to optimize variables at different layers. An optimizer is a function or an algorithm that modifies the attributes of the neural network, such as weights and learning rate. Thus, it helps in reducing the overall loss and improving accuracy^73^. The most commonly used optimization algorithms for this purpose are Stochastic Gradient Descent (SGD), Adaptive Moment Optimization (Adam), Root Mean Square Propagation (RMSProp), SGD + Momentum, Adagrad, and Adadelta. We used ADAM to optimize the results of the DL-CNN models introduced by^74^. In addition to replacing SGD, ADAM takes advantage of AdaGrad and RMSprop, which perform better on sparse gradients and unstable conditions, respectively^74^. Equations (7) and (8) defined the ADAM optimizer:

7 $${m}_{t}^{(j)}={\beta }_{1}{m}_{t-1}^{(j)}+(1-{\beta }_{1}){g}_{t}^{(j)}$$

8 $${v}_{t}^{(j)}={\beta }_{2}{v}_{t-1}^{(j)}+(1-{\beta }_{2})({g}_{t}^{(j)}{)}^{2}$$

where ${m}_{t}^{(j)}$ and ${v}_{t}^{(j)}$ are moving averages, ${g}_{t}^{(j)}$ is gradient on current mini-batch, ${\beta }_{1}$ and ${\beta }_{2}$ are commonly chosen to be 0.9 and 0.999, respectively^75^. The first and second moments are then bias-corrected^76^:

9 $${\widehat{m}}_{t}^{(j)}=\frac{{m}_{t}^{(j)}}{1-{\beta }_{1}^{t}}, {\widehat{v}}_{t}^{(j)}=\frac{{v}_{t}^{(j)}}{1-{\beta }_{2}^{t}}$$

where ${\widehat{m}}_{t}^{(j)}$ is moment.

And used to weight the update:

10 $${w}_{t+1}^{(j)}={w}_{t}^{(j)}-\frac{\alpha }{\sqrt{{\widehat{v}}_{t}^{(j)}+\varepsilon }} {\widehat{m}}_{t}^{(j)}$$

where $\alpha$ is the initial learning rate, which the default value for it is 0.001 and ${w}_{t+1}^{(j)}$ is weights of variables.

### Remote sensing-based approach for identifying frost-affected areas

**Figure 3:**
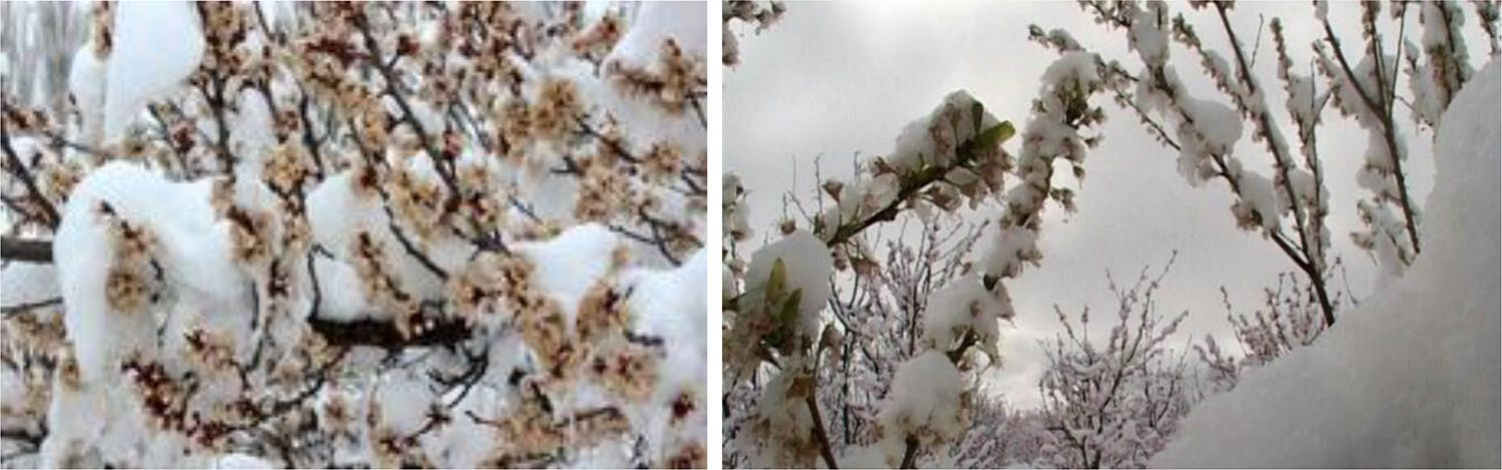
Examples of frost-affected trees, collected from the study area using camera.

Frost is a special weather condition where the ambient temperature drops below the minimum temperature for plant growth and development^77^. In meteorology, freezing occurs when the air inside a meteorological shelter reaches zero or below zero degrees Celsius. In contrast, frostbite does not necessarily occur at temperatures below zero, and the damage occurs at temperatures above zero^78^. Agricultural products can grow in a temperature range (Table 2). The growth of the plants will be reduced if the temperature deviates from this range. The products may even be lost completely if the temperature deviates from this range. As soon as the air temperature falls below the freezing threshold of plants, the interstitial water of the plants freezes, causing the tissue to disintegrate^79^. Generally, there are two types of frost, transitional and radiation. In transitional frost, cold air passes through a region and is replaced by warm air so that its temperature falls below or equals the critical temperature for specific plants. The wind makes this type of frost extremely cold, which is one of its characteristics. As a result, plants lose heat quickly. This phenomenon usually occurs at high altitudes or in lowlands and swamps^77^. On the other hand, radiation frost occurs under stable weather conditions and clear nights, without air mass movement. Because the soil and plants lose heat through radiation, the air near the ground becomes colder due to contact with these cold surfaces, and as a result, plants also become cold due to contact with these cold surfaces^79^. Figure 3 shows examples of frost-affected trees. For frost-affected areas detection, this study employed ANP, which will be explored in the following section:

**Table 2:** Critical temperature (°C) of fruit trees in different stages of growth.

Fruit types	Bud stage	Flowering stage	Fruit stage	Time (m)
Apple	− 4 to − 7.2	− 1.5 to 3	− 1.3 to 1.5	30 to 60
Pear	− 3 to − 2.3	− 2.3 to − 1.5	− 1	30 to 60
Peach	− 5 to − 4	− 2.7 to − 1.3	− 2.3	30 to 60
Cherry	− 6 to − 1.5	− 2.3 to − 1.3	− 1.5	30 to 60
Tomato	− 6.5 to − 1.3	− 1.3 to 0.5	0.5	30 to 60
Apricot	− 4 to 1.3	− 2 to − 0.5	0	30 to 60
Plum	− 4 to − 1.5	− 1.5 to − 0.5	− 1.3 to − 0.5	30 to 60
Almonds	− 4.5	− 2.7	− 1.3	30 to 60
Grape	− 1.3	− 0.5	− 0.5	30 to 60
Walnut	− 1	− 1	− 1	30 to 60

### Analytical Network Process (ANP)

Analytical networks are widely used in modeling analysis and most frequently used in Geographic Information System and Multi-Criteria Decision Analysis (GIS-MCDA)^80^. This is a variation of the analytical hierarchical process (AHP), which was developed for network methods to reduce errors. It is more efficient to use an ANP model since it identifies possible dependencies among the selected criteria^81^. An ANP model articulates its decision-making process through clusters and nodes. Models based on ANP offer a logical way of dealing with dependence and are capable of resolving complex issues involving interdependent relationships. In order to derive the pairwise comparison matrix, we can use the pairwise comparison matrix provided in Table 3 and grade the significance of factors on a scale of 1–9^81^.

**Table 3:** Pairwise comparison point-based rating scale of ANP.

Importance level	Ranking
Equally important	1
Equally crucial to slightly more important	2
Slightly more important	3
Slightly more important to much more important	4
Much more important	5
Much more critical to very much more important	6
Very much more important	7
Very much more important to extremely importance	8
Extremely importance	9

The ANP constructs a network consisting of small to large matrices of elements and probable substitutes grouped into clusters. Within the network, these elements can be intertwined in many ways. The network consists of feedback and interdependence connections within and between clusters^82^.

Priority is calculated separately for each pairwise comparison matrix. To determine the final weighting of the elements, the results of all pairwise comparison matrices are compiled into a super-matrix^68^. Within the network system, the super-matrix W illustrates how elements influence each other. Clusters are formed from small to large matrices of criteria and 11 probable substitutes in the ANP. The network can intertwine these substitutes or alternatives in various ways, containing interdependencies and feedbacks within and between clusters^83^. For the evaluation by the experts, an underlying scale is proposed for pairwise comparisons in the AHP model where values range from 1 to 9. Table 3 provides this ranking. Table 4 shows the cluster matrix based on the ANP for frost-affected areas mapping. LST, sunny days, and precipitation were prioritized as the first, second and third variables with the weights of 0.282, 0.198, and 0.152, respectively for frost-affected areas mapping from 2002 to 2021 as shown in Table 4.

**Table 4:** Various predisposing variables for frost-affected areas mapping and their weights using the ANP.

Criteria	Weight
	2002, 2010, 215, and 2021
LST	0.282
Sunny days	0.198
Precipitation	0.152
Cloud ratio	0.088
DEM	0.085
Soil moisture	0.076
Aspect	0.051
Slope	0.045
Soil type	0.023

### Prediction of the impacts of climate change on the FS using the CA–Markov method

Markov chain is a model which expresses state changes using the transfer matrix from one state at the time ${t}_{1}$ to another at a time ${t}_{2}$. Using this method, the next state will depend on the current state, the previous states, and the neighbors^84^. Theoretically, CA–Markov prediction models have four components: cellular space, cellular state, neighborhood, and transition rules. The cellular space represents the grid area of the whole targeted research area. The range of cellular states, including neighborhoods, is a pixel space matrix of LULC type, with the transformation rule representing the mathematical function of cellular change, i.e., the driving factors behind LULC type change^84,85^.

This study used the CA–Markov model in IDRISI 17.0 Selva software to predict agricultural lands spatial patterns in the LUB. The classified images of the AL pattern in 2002 and 2021 were used for preparing the status conversion matrix. For verification, the Kappa coefficient was used. Table 5 reveals the accuracy of the CA–Markov model for predicting the AL pattern in the LUB. As we see from Table 5, the CA–Markov model with the Kappa coefficient of > 0.8800 performs well for forecasting the pattern of AL and frost-affected areas.

**Table 5:** Accuracy of the CA–Markov method for predicting the AL pattern and frost-affected areas in the LUB.

Years	AL	Frost
2030	0.8800	0.8970
2040	0.8947	0.8835
2050	0.8896	0.8808
2060	0.8912	0.8878

### Accuracy assessment

#### Validation of AL and frost-affected areas classification

In order to assess the accuracy of the DL-CNN agricultural land classification approach and frost modeling approach, intersection over union (IOU) and accuracy (ACC) were used, which are defined by Eqs. (11) and (12), respectively. Table 6 also indicates the results of DL-CNN and remote sensing-based approach for AL classification and modeling frost-affected areas. As we see in Table 6, DL-CNN AL models performed well with the ACC of 0.964, 0.972, 0.975, and 0.971 for the years 2002, 2010, 2015, and 2021, respectively. The results of the remote sensing-based approach in conjunction with the ANP also indicate the ACC of 0.935, 0.925, 0.938, and 0.934 for frost-affected areas mapping for the years 2002, 2010, 2015, and 2021, respectively.

11 $$IOU=\frac{AO\bigcap EO}{AO\bigcup EO}=\frac{TP}{TP+FP+FN}$$

12 $$Accuracy=\frac{TP+TN}{TP+TN+FN+FP}$$

where $AO$ denotes actual output; $EO$ denotes on behalf of expected result; $TP$, $FP$, $FN$, and $TN$ reveal true positive, false positive, false negative, and true negative, respectively.

**Table 6:** Results of accuracy assessment for AL classification and frost-affected areas using IOU and ACC indexes.

Years	AL		Frost	
	IOU	ACC	IOU	ACC
2002	0.846	0.964	0.835	0.935
2010	0.857	0.972	0.938	0.925
2015	0.859	0.975	0.944	0.938
2021	0.860	0.971	0.941	0.934

### Study area

**Figure 4:**
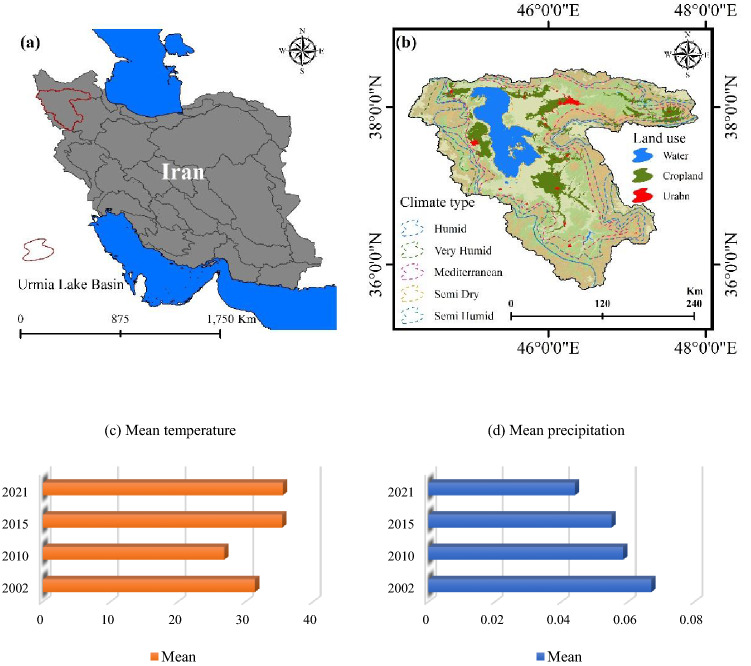
Location of the LUB, (**a**) in the north-western part of Iran. (**b**) Climate types and LULC of the LUB, generated in the ArcGIS 10.6 software (www.esri.com). (**c**,**d**) Are the mean temperature (°C) and precipitation (mm) of the study area from 2002 to 2021, respectively, generated in Microsoft Excel 2022 software (www.microsoft.com).

The study area was LUB, which is located in northwest Iran (Fig. 4). It covers 51,951 km^2^ and ranges in elevation from 1202 m at the lake bed to 3751 m in the Sahand Mountains. Semi-arid climate conditions prevail in the study area. LUB includes Urmia Lake, the primary irrigated agricultural area of Urmia, some other minor irrigated areas, and wide rain-fed wheat agriculture or rangelands spread across the hillside and mountainous region that extends to the western border of the basin^86^. An average annual temperature of 11.7 °C is recorded at Urmia station (10-year average from 2007 to 2016), with the lowest monthly temperature in January (− 1.9 °C) and the highest in July (24.4 °C). Annual precipitation is 294 mm, with approximately 20–40 mm falling on average each month from October to June, and nearly no precipitation falling between July and September^87^. In the study area, apples are the most commonly irrigated crop. Typically, alfalfa or hay crops are planted beneath apple trees. Recent increases in apple cultivation have been attributed to the lake’s shrinking due to a long irrigation period compared to other primary crops. Grapes, peaches, apricot, wheat, sugar beets, and vegetables are primary irrigated crops. Traditionally, surface and groundwater have been used for irrigation; however, drip irrigation is becoming increasingly popular^88^.

#### Impacts of climate change in the study area

**Figure 5:**
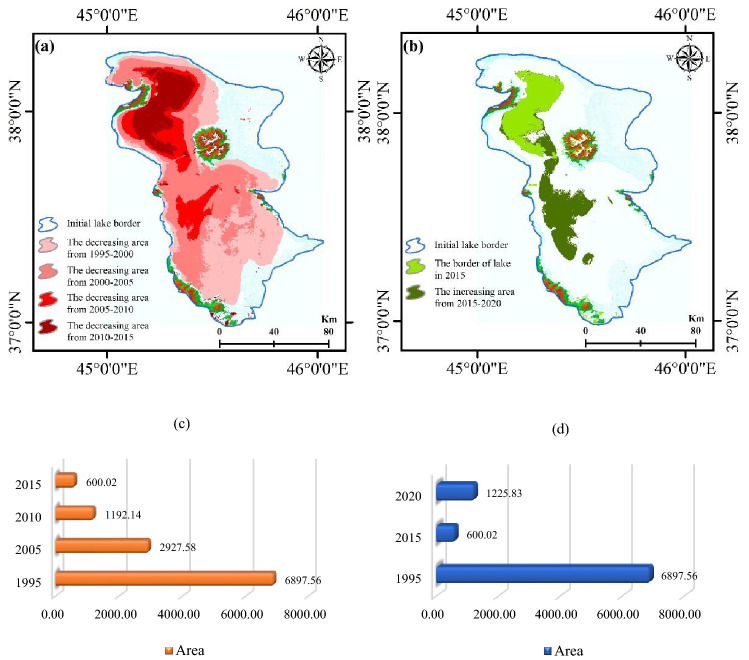
(**a**,**c**) Decreasing (km^2^), and (**b**,**d**) increasing water levels (km^2^) from 1995 to 2020, generated in the ArcGIS 10.6 software (www.esri.com) and Microsoft Excel 2022 software (www.microsoft.com).

As a result of climate change and other factors, such as LULC changes and droughts, there has been a significant change in the water surface area of Lake Urmia in recent years (Fig. 5), especially in the southern areas where the lake bed occasionally exposes during the summer and fall seasons. According to the weather station at Urmia, 2015 had the lowest lake surface area in 2014–2016, while 2016 had the highest. As of 2015 and 2016, the minimum surface area in August was 600.02 km2, and the maximum surface area was 2500 km2. Since^89^ reported 4750–6100 km2 of the lake surface, the lake has shrunk rapidly. Previous studies show that climatic changes and anthropogenic activities have contributed to the current critical situation^3,90–92^. A research study suggests climatic changes are primarily responsible for Lake Urmia shrinkage, and intense water extraction downstream worsens the desiccation process. Thus, Lake Urmia's survival depends strongly on future climate conditions and agricultural water withdrawals^93^.

In LUB, agriculture consumes about 60% of all available water^94^. As result of this trend and other subsidiary effects of climate change, including rapid groundwater depletion, decreased soil fertility, soil erosion and salinization of agricultural lands, and increased water scarcity, as well as threaten the FS.

## Results

### Delineation of AL

**Figure 6:**
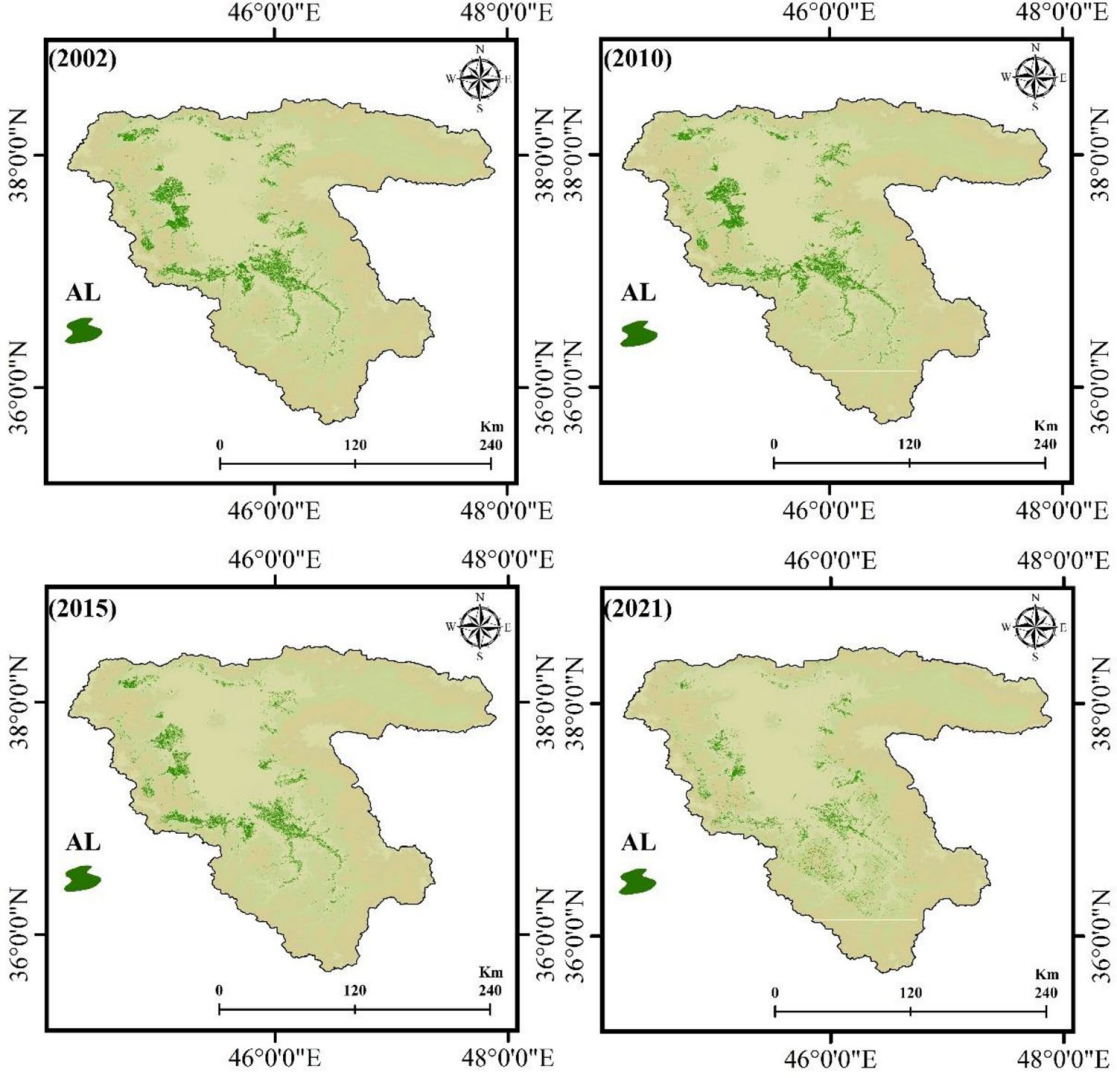
Results of the DL-CNN agricultural land classification method for AL extraction from 2002 to 2021, generated in the ArcGIS 10.6 software (www.esri.com).

**Figure 7:**
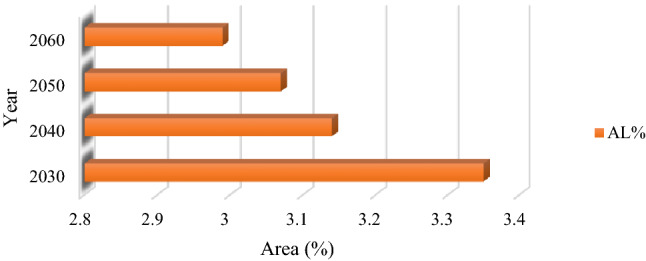
Agricultural lands variation within the LUB form 2002 to 2021, generated in Microsoft Excel 2022 software (www.microsoft.com).

AL plays an important role in modeling climate change's effects on the FS. We used 978 GCPs for AL detection and an automated DL-CNN algorithm. Figure 6 reveals results obtained using a DL-CNN agricultural land classification model based on Landsat series images and GEE. Additionally, Fig. 7 presents information about the areas (%) of AL within the LUB from 2002 to 2021. DL-CNN shows the best performance (> 0.96) for detecting and mapping ALs (Table 6). The LUB has decreased agricultural lands from 3.81 to 3.43%, as shown in Figs. 6 and 7. As mentioned earlier, agriculture is the main food source in the LUB. A reduction in AL would threaten the FS in villages and cities (e.g., Urmia, Tabriz, etc.) within the study area.

### Delineation of frost-affected areas

**Figure 8:**
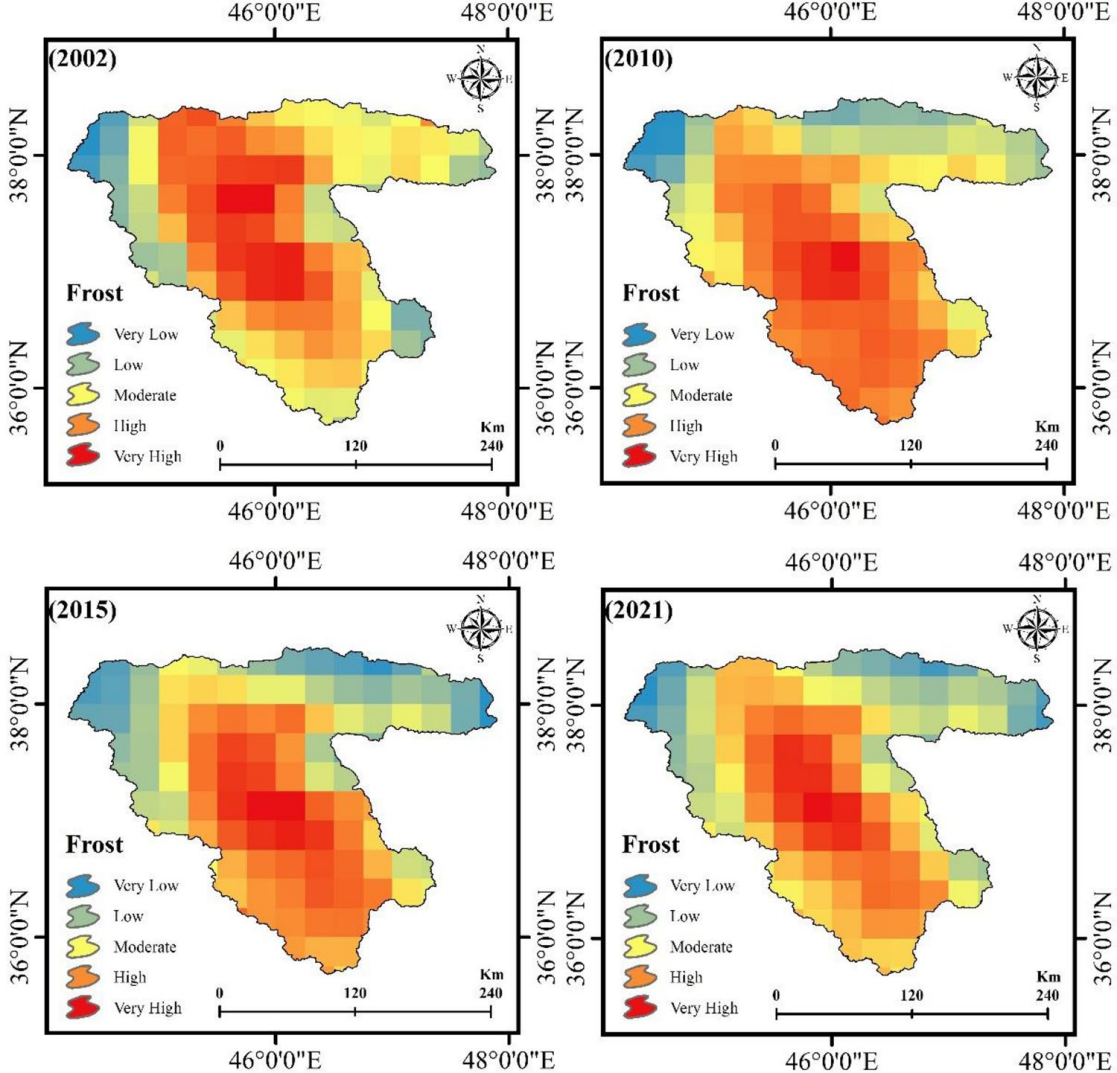
Frost-affected areas in the LUB form 2002 to 2021, generated in the ArcGIS 10.6 software (www.esri.com).

**Figure 9:**
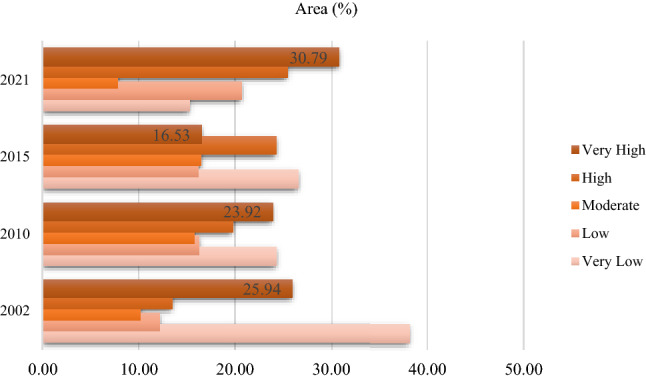
Frost-affected areas variation (%) within the LUB form 2002 to 2021, generated in Microsoft Excel 2022 software (www.microsoft.com).

Additionally, we used remote sensing variables (DEM, aspect, slope, LST, precipitation, and soil moisture), meteorological datasets (sunny days and cloud ratio), and the ANP method to detect and model climate change impacts on the FS based on frost-affected areas. Figure 8 shows the most frost-affected areas in the study area from 2002 to 2021. Our accuracy assessment indicates the satisfying results (> 0.92) of the remote sensing-based technique in concert with the ANP (Table 6). As shown in Fig. 9, the area and percentages of each class are further detailed. Based on Figs. 8 and 9, it can be concluded that frost-affected areas within the LUB increased between 2002 and 2021. In the south and west parts of the LUB, areas where gardens are located, are nearly covered in frost, as shown in Figs. 6 and 8. Within 19 years, the area with very high frost values increased from 25.94 to 30.79% in the study area (Fig. 4).

### Delineation of frost threshold for each garden product

In determining frost-affected areas, LST plays a vital role. The Zonal Statistics Table tool in the Arc GIS environment estimated frost resistance thresholds in Celsius degrees (°C) for apple, peach, grape, and apricot garden products. Table 7 represents the frost resistance thresholds for apple, peach, grape, and apricot, which $*$ means the exact threshold that apple, peach, grape, and apricot have. Table 7 results indicate that apple, peach, and apricot trees suffered the most damage (100%) in 2010 and 2015. In March, when all trees start to bud, most frosts occurred, according to data collected from West and East Azerbaijan Meteorological Organizations (www.eamo.ir). Meteorological data indicate that between 2010 and 2015, the minimum temperature reached − 4.5 °C, which exceeded apple, peach, and apricot resistance thresholds. A frost affected grape trees in the second rank, causing 80% to 90% damage. For example, in 2010, 87,757 ha of the LUB's lands were cultivated with fruit trees, which was expected to yield 824,806 t of crops. According to the Agricultural Organization of West Azerbaijan Province (AOWAP) (www.waaj.ir), more than 58,760 ha of fruit orchards were damaged by frost, and the amount of production decreased to 482, 240 t. According to Table 7, frost damage in 2021 was estimated at 70% to 100%. Accordingly, peach and grape products tolerated the most damage, with frost resistance thresholds of <  − 1.3 and <  − 5 °C, respectively. Apricots and apples were ranked second and third, respectively in 2021. In 2002, fewer trees were affected by frost than in 2010, 2015, and 2021. Approximately 80% to 90% of the damage was estimated this year. The frost resistance thresholds for apple, peach, and apricot trees were between − 1 and − 3 °C. From 2002 to 2021, the impacts of climate change on garden products increased. A sudden snowfall during the last decade caused frost in March over the study area, causing the temperature to drop. The research results over 19 years show that the LUB has a high potential for frostbite. The LUB has frost damage every year, but the amount varies. According to the present study, the frost of garden lands was over 80% in the years under study, which has caused huge economic losses to the government and farmers.

**Table 7:** Frost resistance in Celsius degrees (°C) for each garden products generated using The Zonal Statistics Table tool and their predicated damages.

Years	Fruit types	Very low (> 1)	Low (1 to − 1)	Moderate (− 1 to − 3)	High (> − 3)	Frost percentage
2002	Apple			*		80–90
	Peach			*		
	Grape		*			
	Apricot			*		
2010	Apple				*	100
	Peach				*	
	Grape			*		
	Apricot				*	
2015	Apple				*	100
	Peach				*	
	Grape			*		
	Apricot				*	
2021	Apple			*		70–100
	Peach				*	
	Grape				*	
	Apricot	*				

### Delineation of frost-damage assessment for each class

As shown in Table 8, we estimated frost damage for each class in different products to understand how frost impacts fruit. Table 8 shows that apricot, apple, and peach frost damage was 80%, 40%, and 37%, respectively, in 2002. Additionally, grape products with a damage rate of 5.3% were not significantly damaged in the LUB. Table 8 shows that 100% of apricots, 97% of apples, and 91% of peaches suffered frost damage in 2010. Grape products with a damage rate of 74% were ranked fourth within the LUB. According to Table 8, 100% of apricots, 100% of apples, and 96% of peaches were damaged by frost in 2015. There was also significant damage to grape products with a damage rate of 89% over the study area. Table 8 indicates that 99% of grapes, 92% of peaches, and 38% of apples were damaged by frost in 2021 within the LUB. A damage rate of 3% was also observed for apricot products.

**Table 8:** Frost-damage assessment for each class in different products.

Years	Fruit types	Very low	Low	Moderate	High	Very high
2002	Apple	0	0	0	40%	0
	Peach	0	0	0	37%	0
	Grape	0	0	5.3%	0	0
	Apricot	0	0	0	80%	0
2010	Apple	0	0	0	0	97%
	Peach	0	0	0	0	91%
	Grape	0	0	0	74%	0
	Apricot	0	0	0	0	100%
2015	Apple	0	0	0	0	100%
	Peach	0	0	0	0	96%
	Grape	0	0	0	89%	0
	Apricot	0	0	0	0	100%
2021	Apple	0	0	0	38%	0
	Peach	0	0	0	0	92%
	Grape	0	0	0	0	99%
	Apricot	0	3%	0	0	0

### Prediction of FS maps using the CA–Markov method

**Figure 10:**
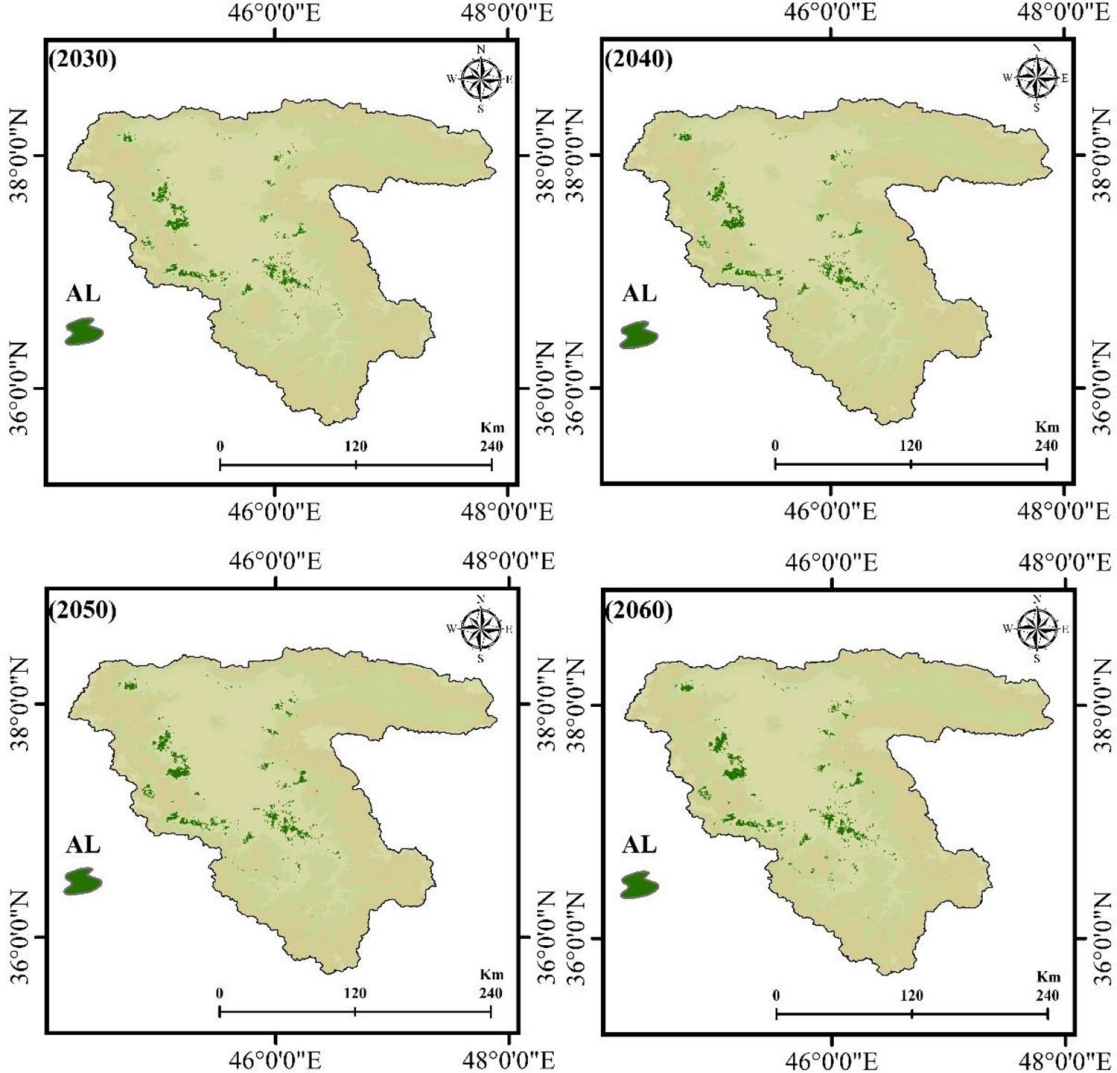
Results of the AL predication using the CA–Markov model for the years 2030, 2040, 2050, and 2060, generated in the ArcGIS 10.6 software (www.esri.com).

**Figure 11:**
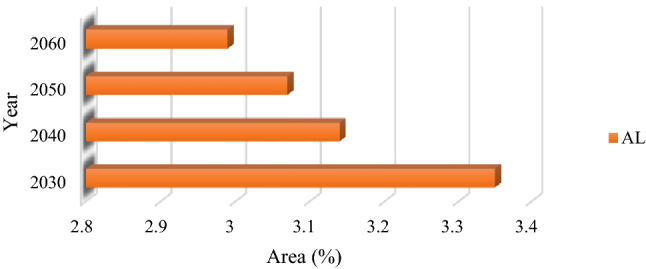
The AL areas variation (%) within the LUB for the years 2030, 2040, 2050, and 2060, generated in Microsoft Excel 2022 software (www.microsoft.com).

**Figure 12:**
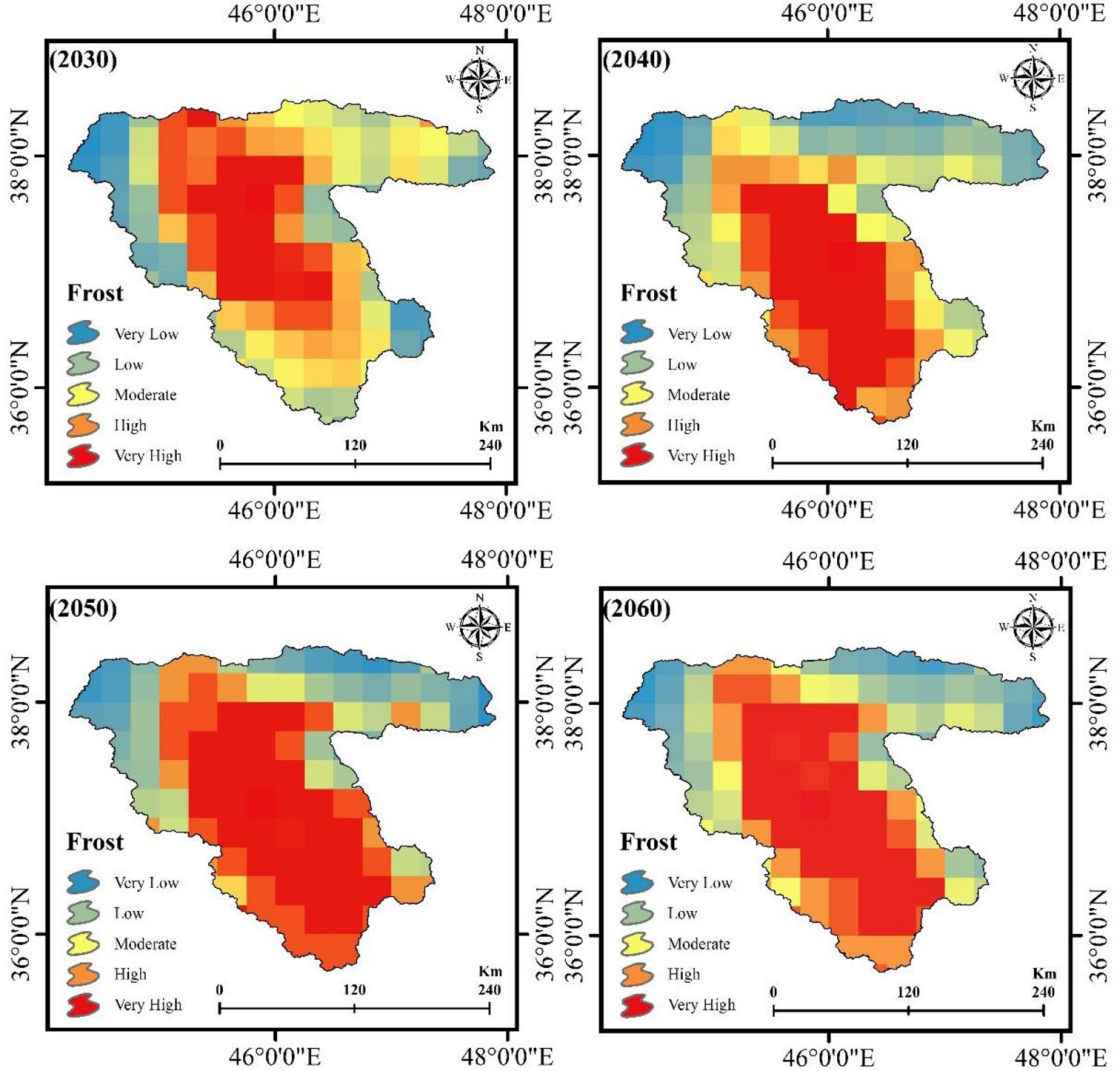
Results of the frost-affected areas predication using the CA–Markov model for the years 2030, 2040, 2050, and 2060, generated in the ArcGIS 10.6 software (www.esri.com).

**Figure 13:**
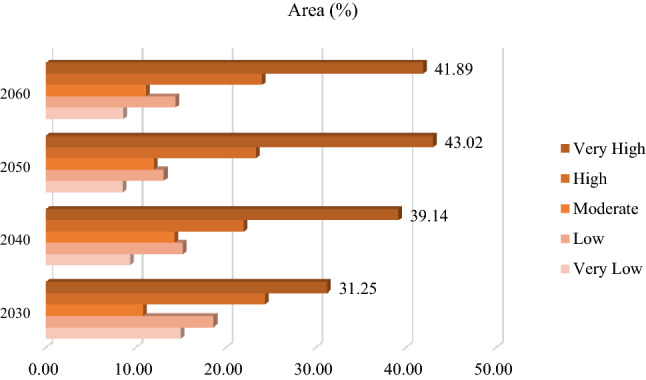
The frost-affected areas variation within the LUB for the years 2030, 2040, 2050, and 2060, generated in Microsoft Excel 2022 software (www.microsoft.com).

After identifying the FS trend from 2002 to 2021, the combined cellular automaton and markov model was used to predict the impacts of climate change on the FS for the years 2030, 2040, 2050, and 2060. Figures 10 and 12 reveal the AL and frost-affected areas simulation results using the CA–Markov for the years 2030, 2040, 2050, and 2060, respectively. The AL and frost-affected areas variations also are presented in Figs. 11 and 13, respectively. According to Figs. 10 and 11, the AL will experience a reduction from 2030 to 2060 in its size. Throughout the LUB, the area of the AL is estimated to be reduced from 3.35 to 2.99% as shown in Fig. 11. With a general look at Figs. 12 and 13, it can be seen an increase by about 10.64% in the area of very high frost-affected from 2030 to 2060. According to Fig. 13, areas with very high frost-affected will be 31.25%, 39.14%, 43.02%, and 41.89% for the years 2030, 2040, 2050, and 2060, respectively. In sum, it can be concluded that the FS threat will be more severe in the future compared to the present time, which requires urgent solutions for this crucial phenomenon in the LUB.

### Impacts of predisposing variables on the FS

FS is influenced by a variety of variables that affect plant growth. As we see from Table 9, the mean values of climatic variables increased within the study area from 2002 to 2021. As shown in Table 9, the mean values of LST were 31.4, 26.85, 35.42, and 35.5 °C for 2002, 2010, 2015, and 2021, respectively. With a simple subtraction, it can be stated that the mean value of LST increased by about 4.1 $^\circ$ C in 2021 compared to 2002. A significant increase (8.57 °C) is also visible in 2015 compared to 2010. The highest drying of Lake Urmia this year would be responsible for this phenomenon, which lands with very high salinization increased from 18.69 to 22.28% within the LUB (Table 9). In addition, a reduction in vegetation cover throughout the study area can be another possible reason for the LST increase.

**Table 9:** The min, max and mean values of predisposing variable for FS assessment.

Variable	Value	2002	2010	2015	2021
LST	Min	9.7	1.8	12.9	12.8
	Max	53.1	51.9	57.94	58.2
	Mean	31.4	26.85	35.42	35.5
ET	Min	1	1	1	1
	Max	297	289	329	323
	Mean	149	145	165	162
Precipitation	Min	0.04	0.029	0.031	0.018
	Max	0.094	0.088	0.079	0.07
	Mean	0.067	0.0585	0.055	0.044
Sunny days	Min	145.7	143.2	138.9	140
	Max	173.4	170.9	162.2	164.9
	Mean	159.6	157.1	150.6	152.5
Cloud ratio (%)	Min	41	43	48	51
	Max	43	47	56	57
	Mean	42	45	52	54
Soil salinity	High salt (%)	12.1	12.4	13.53	13.56
	Very high salt (%)	1.35	18.69	22.28	21.61
Soil moisture	Min	24	37.5	19.5	15.15
	Max	928	596	323	273
	Mean	476	316.75	171.25	144.075
Groundwater quality	Min	17.2	17.1	23.1	26.2
	Max	6920.1	11,660.1	1345.6	1147.1
	Mean	3468.65	5838.6	684.35	586.65

According to Table 9, the mean value of evapotranspiration (ET) experienced an increase from 2002 to 2021. The mean values of ET were estimated at 149, 145, 165, and 162 ml for the years 2002, 2010, 2015, and 2021, respectively. As we see from Table 9, the mean value of ET increased by about 13 ml from 2002 to 2021. Precipitation is key in balancing the ET value. In this regard, a reduction in precipitation rate, soil moisture as well, and other factors increasing, such as LST, can be highlighted for ET increase over the LUB (Table 9).

A general look at Table 9 shows that the precipitation rate was reduced from 2002 to 2021. The mean value of precipitation was 0.067 in 2002. This rate decreased to 0.044 (0.023) in 2021, coinciding with meteorological stations’ reports (http://eamo.ir).

According to Table 9, the mean numbers of sunny days were 159.6, 157.1, 150.6, and 152.5 for 2002, 2010, 2015, and 2021, respectively. With a simple calculation, it can be obtained that the mean number of sunny days reduced to about 7.1 days from 2002 to 2021. The increase in the percentage of cloud ratio within the study area would be a possible reason for this.

With a general look at Table 9, it can be seen that the mean days of cloud ratio increased from 2002 to 2021. The mean values of cloud ratio were 42, 45, 52, and 54 days for the years 2002, 2010, 2015, and 2021, respectively. The mean value of the cloud ratio decreased by about 12% between 2002 and 2021, as shown in Table 9.

As we see from Table 9, areas with high and very high soil salinity increased from 2002 to 2021. Table 9 illustrates that areas with high salinity covered 12.1%, 12.4%, 13.53%, and 13.56% of the LUB for the years 2002, 2010, 2015, and 2021, respectively. Additionally, areas with very high salinity covered 1.35%, 18.69%, 22.28%, and 21.61% of the study area for 2002, 2010, 2015, and 2021, respectively. Table 9 shows that areas with high salinity increased by about 1.46% within 19 years. Furthermore, areas with very high salinity increased significantly (20.26%) from 2002 to 2021 (Table 9). Since Lake Urmia has dried up, salt crystals have replaced the water in the lake.

With a general look at Table 9, it can be stated that the mean rate of soil moisture reduced from 2002 to 2021. Table 9 shows that the mean rates of soil moisture were 476, 316.75, 171.25, and 144.075 for the years 2002, 2010, 2015, and 2021, respectively. As shown in Table 9, this value decreased by 331.925 between 2002 and 2021. Increasing LST, ET and decreasing precipitation would mainly be responsible for this phenomenon.

Table 9 shows groundwater quality was significantly reduced from 2002 to 2021. In this regard, the mean values of groundwater quality were 3468.65, 5838.6, 684.35, and 586.65 for the years 2002, 2010, 2015, and 2021, respectively as shown in Table 9. With a simple subtraction, it can be stated that the mean value of groundwater quality decreased by about 2882 from 2002 to 2021, which is a remarkable number. Increased number of deep and semi-deep wells (See Fig. 1h) and shortage of precipitation would be the main reasons for this reduction.

### Frequency Ratio (FR)

Using the FR model, a simple tool for assessing the probabilistic relationships between dependent and independent variables can be presented^95^. The mentioned approach can be stated as an FR index representing the quantitative relationship between AL and frost-affected areas and different conditioning factors. It can be expressed based on Eq. (13)^96^. Tables 10 and 11 also illustrate the efficiency of different variables for AL and frost-affected area modeling and mapping using FR. Based on Tables 10 and 11, precipitation, groundwater quality and LST show the highest potential for food security assessment over the study area from 2002 to 2021.

13 $$FR=\frac{E/F}{M/L}$$

where $E$ is the number of pixels with AL and frost-affected area for each variable, $F$ is the total number of AL and frost-affected area in the study area, $M$ is the number of pixels in the class area of the variable and $L$ is the number of total pixels in the study area.

**Table 10:** Efficiency of different variables for AL modelling and mapping using the FR.

Efficient variables in 2002	FR	Efficient variables in 2010	FR	Efficient variables in 2015	FR	Efficient variables in 2021	FR
Precipitation	0.9728	Precipitation	0.9503	Groundwater quality	0.9623	Groundwater quality	0.9689
Groundwater quality	0.9633	Groundwater quality	0.9141	Precipitation	0.9499	Precipitation	0.9547
ET	0.9350	ET	0.8880	ET	0.9233	ET	0.9214
Soil moisture	0.9028	Soil moisture	0.8618	Soil moisture	0.9158	Soil moisture	0.9002
LST	0.8778	LST	0.8662	Soil salinity	0.8925	Soil salinity	0.8875
Sunny days	0.8236	Soil salinity	0.8597	LST	0.8725	LST	0.8542
Cloud ratio	0.7715	Sunny days	0.8321	Sunny days	0.8169	Sunny days	0.8269
Soil types	0.7125	Cloud ratio	0.7583	Cloud ratio	0.7768	Cloud ratio	0.7635
Slope	0.6421	Soil types	0.6702	Soil types	0.6958	Soil types	0.7015
Aspect	0.6058	Slope	0.5936	Slope	0.6324	Slope	0.6458
DEM	0.5289	Aspect	0.5547	Aspect	0.5632	Aspect	0.5798
Soil salinity	0.5017	DEM	0.5231	DEM	0.5047	DEM	0.4725

**Table 11:** Efficiency of different variables for frost-affected area modelling and mapping using the FR.

Efficient variables in 2002	FR	Efficient variables in 2010	FR	Efficient variables in 2015	FR	Efficient variables in 2021	FR
LST	0.9636	LST	0.9763	LST	0.9868	LST	0.9895
Sunny days	0.9520	Sunny days	0.9489	Sunny days	0.9542	Sunny days	0.9643
Precipitation	0.9204	Precipitation	0.9275	Cloud ratio	0.9364	Precipitation	0.9455
Cloud ratio	0.8967	Cloud ratio	0.9175	Precipitation	0.9012	Cloud ratio	0.9018
DEM	0.8725	DEM	0.8962	DEM	0.8857	DEM	0.8928
Soil moisture	0.8699	Soil moisture	0.8596	Soil moisture	0.8754	Soil moisture	0.8712
Aspect	0.8578	ET	0.8411	ET	0.8512	Aspect	0.8843
ET	0.8345	Aspect	0.8014	Aspect	0.8145	ET	0.8217
Slope	0.8032	Soil types	0.7802	Soil types	0.7935	Slope	0.8186
Soil type	0.7812	Slope	0.7626	Slope	0.7785	Soil type	0.7898
Groundwater quality	0.7058	Groundwater quality	0.6758	Groundwater quality	0.5436	Groundwater quality	0.5678
Soil salinity	0.6458	Soil salinity	0.6102	Soil salinity	0.4785	Soil salinity	0.5101

## Discussion

### General discussion

Hunger is increasing worldwide. Following 2 decades of a downward trend, the number of hungry people in the world underwent a drastic change in the early 1990s. Since then, it has increased due to the rise in food prices. The financial crisis, in turn, caused the price of agricultural products to fall to some extent and the trade of agricultural products and related exchanges decreased. Today, many groups of hungry people in the world are directly or indirectly dependent on agriculture for their survival. According to the latest assessment carried out by the Food and Agriculture Organization (FAO), today more than 969 million people in the world live on less than one dollar a day, and about three-quarters of them depend on agriculture for their survival. Today, the impact of climate on the agricultural sector is one of the most important environmental challenges of the twenty-first century, which has serious economic consequences and affects various sectors, including agriculture, forestry, water, industry, tourism and insurance. It affects undoubtedly, one of the effective factors in the economic development of any country is directed to its agriculture and horticulture field, which is considered to be the provider of health and food independence for every nation. Therefore, in the event of failure and disruption in this area, one of the main and fundamental pillars of economic development will be weakened and shaken. To increase food security, appropriate methods (e.g., remote sensing-based methodology) are necessary. In addition to providing satellite images of different spatial and temporal resolutions, remote sensing allows timely management of land uses, which increases the power of decision-making, optimized exploitation, and more precise agricultural planning. In this study, we employed an integrated approach of remote sensing, GEE, DL-CNN and CA–Markov to evaluate the impacts of climate change on the FS and predict its impacts for the future. Our study found that the integrated approach has the greatest potential for monitoring the FS. In addition, the results of this study illustrate the significant impacts of climate change on the FS. Figures 6, 7, 8, and 9 show how climate change affected the FS in the LUB by decreasing agricultural lands (from 3.81% in 2002 to 3.43% in 2021) and increasing frost-affected areas from 25.94 to 30.79% between 2002 and 2021.

### Coefficient correlation analysis

The results of correlation analysis to identify the relationship between predisposing variables and AL and frost-affected areas for the FS assessment are shown in Table 12. Table 12 shows a strong negative correlation of − 0.80 between AL and LST. This means that in the LUB, the increase in LST is associated with a decrease in AL. Table 12 shows a negative correlation coefficient of − 0.58 between AL and ET. In this regard, the increase in ET leads to the reduction of AL. In addition, we found negative correlations of − 0.43 and − 0.0.45 between AL and cloud ratio and soil salinity, respectively as shown in Table 12. The increase in cloud ratio and soil salinity leads to the reduction of AL. According to Table 12, positive relationships between AL and precipitation (0.39), sunny days (0.25), soil moisture (0.21), and groundwater quality (0.77) can be seen. In this regard, it can be stated that the decrease in precipitation, sunny days, soil moisture, and groundwater quality is associated with the decrease in AL.

**Table 12:** Correlation coefficient analysis between AL, frost-affected areas and predisposing variables.

	Variable	Correlation coefficient		Variable	Correlation coefficient
AL	LST	− 0.80	Frost	LST	0.55
AL	ET	− 0.58	Frost	ET	0.40
AL	Precipitation	0.39	Frost	Precipitation	− 0.68
AL	Sunny days	0.25	Frost	Sunny days	− 0.23
AL	Cloud ratio	− 0.43	Frost	Cloud ratio	0.52
AL	Soil salinity	− 0.45	Frost	Elevation	0.35
AL	Soil moisture	0.21	Frost	Soil moisture	− 0.38
AL	Groundwater quality	0.77	Frost	Slope	0.45
			Frost	Aspect	0.39

The results of correlation analysis show negative correlations of − 0.068 and − 0.38 between frost-affected areas and precipitation and soil moisture, respectively as shown in Table 12. From these negative numbers, the conclusion is drawn that the decrease in precipitation and soil moisture is associated with the increase in frost-affected areas. With a general look at Table 12, positive correlation coefficients of 0.55, 0.40, 0.52, 0.35, 0.45, and 0.39 can be seen between frost-affected areas and LST, ET, cloud ratio, elevation, slope and aspect, respectively. In this regard, it can be stated that the increase in LST, ET, cloud ratio, elevation, slope and aspect is associated with the increase in frost-affected areas.

### Analysis of the efficiencies of different predisposing variables on the FS using the FR

Table 10 shows precipitation with the FR of 0.9728% for 2002 and 0.9503% for 2010, groundwater quality with the FR of 0.9623% for 2015 and 0.9689% for 2021, showing a high efficiency for food security assessment based on AL. In this regard, it can be stated that the reduction in precipitation ratio and groundwater quality from 2002 to 2021 (Table 9) would be responsible for agricultural land degradation, affecting the FS within the LUB. As we see from Table 10, groundwater quality with the FR of 0.9633% and 0.9141% and precipitation with the FR of 0.9499% and 0.9547% was chosen as efficient factors for the FS assessment for 2002, 2010, 2015, and 2021, respectively. ET with the FR of 0.9350%, 0.8880%, 0.9233%, and 0.9214% was ranked third for the FS evaluation for the years 2002, 2010, 2015, and 2021, respectively, as shown in Table 10. These variables are followed by soil moisture, LST, sunny days, cloud ratio, soil types, slope, aspect, DEM, and soil salinity for the year 2002, soil moisture, LST, soil salinity, sunny days, cloud ratio, soil types, slope, aspect, and DEM for the year 2010, soil moisture, soil salinity, LST, sunny days, cloud ratio, soil types, slope, aspect, and DEM for the year 2015, and soil moisture, soil salinity, LST, sunny days, cloud ratio, soil types, slope, aspect, and DEM for the year 2021 (Table 10).

For food security assessment based on frost-affected areas, LST with the FR of 0.9636%, 0.9763%, 0.9868%, and 0.9895% illustrates the highest potential for 2002, 2010, 2015, and 2021, respectively, as shown in Table 11. As mentioned before, LST is an essential factor for frost assessment, and a small change in its value can affect the plants significantly. With a general look at Table 11, it can be explained the efficiency of LST for frost occurrence. As we see from Table 11, the sunny day with the FR of 0.9520%, 0.0.9489%, 0.9542%, and 0.9643% was chosen as an efficient factor for the FS assessment for the years 2002, 2010, 2015, and 2021, respectively. According to Table 11, precipitation with the FR of 0.9204% for the year 2002 and 0.9275% for the year 2010, cloud ratio with the FR of 0.9364% for the year 2015, and precipitation with the FR of 0.9455% for the year 2021 show a high efficiency for food security assessment based on frost-affected areas. In the following ranks, we have cloud ratio, DEM, soil moisture, aspect, ET, slope, soil types, groundwater quality, and soil salinity for the year 2002, cloud ratio, DEM, soil moisture, ET, aspect, soil types, slope, groundwater quality, and soil salinity for the year 2010, precipitation, DEM, soil moisture, ET, aspect, soil types, slope, groundwater quality, and soil salinity for the year 2015, and cloud ratio, DEM, soil moisture, aspect, ET, slope, soil types, groundwater quality, and soil salinity for the year 2021 as shown in Table 11.

### Analysis of the effects of the FS on local environment and inhabitation

Food insecurity is often associated with malnutrition, in which a person's energy and/or nutrients intake is deficient, excessive, or imbalanced. When an individual's habitual food consumption is insufficient to provide the necessary amount of dietary energy to maintain a normal, active, healthy lifestyle, they are considered undernourished. There is also nutritional undernourishment in terms of nutritional deficiencies in vitamins (e.g., vitamin A) and minerals (e.g., iron, zinc, and iodine), that is, hidden hunger.

According to Shared Socio-economic Pathways (SSPs) 1, 2, and 3, climate change will result in 1–29% cereal price increases in 2050 (RCP 6.0), affecting consumers globally through higher food prices. Low-income consumers are particularly at risk, with models projecting increases of 1–183 million additional people at risk of hunger across the SSPs compared to a no climate change scenario. While CO2 increase is expected to boost crop productivity at lower temperatures, it is expected to lower nutritional quality (e.g., wheat grown at 546–586 ppm CO2 has 5.9–12.7% fewer protein, 3.7–6.5% fewer zinc, and 5.2–7.5% fewer iron). Distributions of pests and diseases will change, affecting production negatively in many regions^44,45^.

According to this study, degrading environmental conditions in the LUB will negatively affect the FS. It has been found that water resources have been mismanaged, agricultural farmlands have been extended, and aquifers have been overdrawn, resulting in a lake drought, as well as extensive land degradation and excessive salinity, which threaten the FS. A lack of sustainable development strategies and mismanagement of the fragile ecosystem have led to these problems over the past 3 decades.

## Conclusion and outlooks

This study employed an integrated framework of remote sensing, GEE and DL-CNN to track climate change’s impacts on the FS. Results reveal a significant change in climatic (e.g., LST) and geospatial (e.g., soil salinity) variables from 2002 to 2021. The results of this study demonstrate the highest performance of automated DL-CNN (ACC of > 0.9600) for AL classification. Such an automated approach is more efficient when combined with GEE and remote sensing datasets. Our findings also reveal the efficiency of a remote sensing-based approach in concert with the ANP (ACC of > 0.9200) for frost-affected areas detection. According to the study’s results, the CA–Markov worked well in predicting the impacts of climate change on the FS for the years 2030, 2040, 2050, and 2060. In sum, results established that computer-based methodologies such as DL-CNN in combination with GEE, remote sensing, and CA–Markov could be used for the FS assessment. Our findings further emphasize the impact of climate change on Lake Urmia, whose water level has been decreasing since 1995 according to the international United Nations Environment Program (UNEP) and the Global Environmental Alert Service (GEAS) reports. Such reduction has led to several ecological problems such as salt storms, soil salinization and groundwater salinization from a lake that is regarded as one of the hyper-saline lakes in the world. The salty lands have been exposed to 8 billion tons of salt, which poses a serious threat to the ecosystems of the ULB and north-west Iran. There is widespread recognition that the Urmia Lake drought seriously threatens the environment, human health, the local economy, and food security of near countries such as Azerbaijan, Turkey and Iraq. In the future, combined effects may result in rural populations migrating and dispersing to other regions of the country. Consequently, LUB faces a very critical environmental situation, and in February 2012, the United Nations Environment Program declared Lake Urmia's status as "worrying," urging special attention and immediate restoration efforts to prevent environmental disasters.

Measures for inputs and activities could already be reported with relatively little development, but more data is needed to develop measures that are more relevant to climate change impacts. Climate change adaptation can also be measured using a range of ecological indicators. Despite the difficulty of developing these indicators, which sometimes require a deeper understanding of climate change impacts and adaptation interventions, it will be much easier to attribute interventions to outcomes. We hope this framework and an open discussion of the challenges associated with its implementation will contribute to the development of urgently needed methods to determine whether our climate change interventions are effective, given the current debate over the precise nature of future technological progress toward sustainable development.

## Data Availability

The datasets that support the findings of this study are available from the corresponding author on reasonable request.
